# Study of the Cytotoxic Effects of Au@Rh Core–Shell Metal Particles on the Osteosarcoma Cell Line HOS and the hFOB Osteoblast Cell Line

**DOI:** 10.3390/ijms27146253

**Published:** 2026-07-14

**Authors:** Sergio Zamudio-Lucero, Martín Trejo-Valdez, Nury Pérez-Hernández, Ángel Bañuelos-Hernández, María Elena Manríquez-Ramírez

**Affiliations:** 1 Unidad Profesional Interdisciplinaria de Biotecnología (UPIBI), Instituto Politécnico Nacional (IPN), Av. Acueducto, La Laguna Ticomán, Gustavo A. Madero, Ciudad de México 07340, Mexico; szamudiol@ipn.mx; 2 Escuela Superior de Ingeniería Química e Industrias Extractivas (ESIQIE), Instituto Politécnico Nacional (IPN), Zacatenco, Edificio 8 1er. Piso, Ciudad de México 07300, Mexico; mmanriquez@ipn.mx; 3 Escuela Nacional de Medicina y Homeopatía (ENMH), Instituto Politécnico Nacional (IPN), Av. Guillermo Massieu Helguera 239, La Escalera, Gustavo A. Madero, Ciudad de México 07320, Mexico; nperezh@ipn.mx (N.P.-H.); abanuelosh@ipn.mx (Á.B.-H.)

**Keywords:** core–shell nanoparticles, cytotoxicity, osteosarcoma cells

## Abstract

Osteosarcoma, the most common primary malignant bone tumor in adolescents, faces treatment challenges due to metastasis and chemoresistance. This study developed a novel Au@Rh core–shell nanoparticle system functionalized with indocyanine green (ICG) to overcome hypoxia-limited photodynamic therapy (PDT). Au@Rh nanoparticles were synthesized via wet chemistry and characterized by UV-Vis spectroscopy, TEM, and cyclic voltammetry (CV). The system exhibited a core–shell morphology, well-defined crystalline planes, photothermal conversion and electrocatalytic activity. The Au@Rh nanoparticles (109 nm total size, 90 nm Au core, and 15 nm Rh shell) demonstrated dual functionality: the gold core provided photothermal conversion (a 7 °C temperature increase under NIR irradiation), while the rhodium shell exhibited pH-independent electrocatalytic activity for H_2_O_2_ decomposition, generating oxygen to alleviate tumor hypoxia. Crucially, the system showed excellent biocompatibility, with no significant cytotoxicity in both osteosarcoma (HOS) or normal osteoblast (hFOB) cells after 48 h of exposure. When activated by NIR irradiation (808 nm, 16.6 J/cm^2^), the complete Au@Rh-ICG system achieved selective 67% cytotoxicity in HOS cells versus only 30% in hFOB cells, demonstrating targeted therapeutic efficacy. These results position Au@Rh-ICG as a promising theranostic platform for osteosarcoma treatment, combining enhanced PDT with photothermal therapy while addressing tumor hypoxia.

## 1. Introduction

Osteosarcoma (OS), the most common primary malignant bone tumor, predominantly affects children and adolescents [1,2]. Standard treatment combines surgical resection with chemotherapy; however, recurrence, metastasis, and chemoresistance remain major clinical challenges [3,4,5,6]. Approximately 25% of patients present with pulmonary metastases at diagnosis, reducing the 5-year survival rate to less than 25% [2]. Genetically, OS is characterized by high genomic instability, frequently involving alterations in the tumor suppressor genes TP53 and RB1 [7]. According to the Global Burden of Disease (GBD) 2021 study, there were an estimated 91,380 new cases of malignant bone and articular cartilage neoplasms worldwide in 2021, with a cumulative prevalence of 598,640 confirmed cases. In the same year, these neoplasms resulted in 66,114 deaths. The disability-adjusted life years (DALYs), a comprehensive measure of overall disease burden, reached 508,202 for the population aged 65 years and older alone [8,9]. In children and adolescents, the incidence rate is as high as 8.7 per million in the United States, and overall survival for patients with metastatic or recurrent disease remains disappointingly low at approximately 20–30% [10]. These quantitative data underscore the substantial global health impact of osteosarcoma and highlight the urgent need for innovative therapeutic strategies.

In addition, OS cells exhibit metabolic reprogramming associated with the Warburg effect, generating an acidic and hypoxic tumor microenvironment that may contribute to tumor progression and therapeutic resistance. Paradoxically, this metabolic reprogramming occurs despite the presence of functional mitochondria [11,12,13,14,15]. The resulting acidification of the tumor niche, in turn, promotes resistance to anoikis (a mechanism enabling cell survival upon detachment from the extracellular matrix), thereby facilitating invasion and metastasis processes [16]. Since the clinical manifestations of osteosarcoma are often ambiguous until advanced stages, therapeutic success critically depends on early diagnosis and targeted treatments, driving the continuous search for therapeutic alternatives and the identification of new molecular targets.

Photodynamic Therapy (PDT) has emerged as a promising adjuvant strategy to overcome conventional resistance in osteosarcoma [17,18,19,20]. This therapy uses photosensitizers (PS) that, when activated by light of a specific wavelength, generate reactive oxygen species (ROS) to induce cell death via oxidative stress [21]. However, the efficacy of PDT is severely limited by the hypoxic tumor microenvironment (pO_2_ ≤ 2.5 mmHg), which restricts the availability of molecular oxygen, an indispensable substrate for ROS generation [22,23].

Advances in nanotechnology offer innovative strategies to improve photodynamic therapy, particularly through the development of nanocarriers and multifunctional nanoparticles. Gold nanoparticles have demonstrated potential as safe nanocarriers with photothermal properties under near-infrared (NIR) irradiation, enabling combined photodynamic and photothermal therapy [24,25,26,27]. In particular, gold nanoparticles functionalized with indocyanine green (ICG), a photosensitizer activatable in the NIR spectrum, have shown synergistic therapeutic effects by combining ROS generation with hyperthermia-induced tumor ablation [27]. Moreover, Li et al. (2017) demonstrated that gold-based ICG nanocarriers may help overcome resistance to photodynamic therapy [28].

Gold–rhodium core–shell (Au@Rh) structures represent the convergence of this dual approach, integrating the photothermal properties of gold with the electrocatalytic capacity of rhodium to degrade tumors by the production of H_2_O_2_. This catalytic activity alleviates hypoxia and, synergistically, enhances the efficacy of PDT [29]. It is worth noting that alternative magnetic nanoparticles, such as iron oxide (Fe_3_O_4_)-based systems, have been explored for similar therapeutic applications [30]. However, these magnetic counterparts present inherent limitations, including rapid clearance by the reticuloendothelial system, a tendency to aggregate, and potential long-term toxicity due to poor biodegradability [31]. In contrast, our Au@Rh core–shell platform avoids magnetic field dependency, offers stable photothermal conversion, and provides sustained electrocatalytic oxygen generation without the need for external magnetic guidance. Rhodium-based nanostructures have shown a promising profile, where synthesis with morphological control (such as nanoshells, nanoframes, and porous nanoplates) has achieved excellent biocompatibility and therapeutic efficiency in photothermal applications [32,33,34,35]. Recently, rhodium alloys have been employed as synergistic agents in osteosarcoma treatment [36], reporting encouraging results. The field of nanomedicine shows notable clinical translation, with 15 approved nanodrugs and approximately 80 candidates in 200 active clinical trials, many of which exploit passive targeting strategies mediated by the EPR (enhanced permeability and retention) effect [37,38,39].

In this work, we report the development and evaluation of an Au@Rh core–shell nanostructured system functionalized with indocyanine green (ICG), designed to overcome key limitations of photodynamic therapy in osteosarcoma. The Au@Rh nanoparticles (Au@Rh NPs) were successfully synthesized via soft-chemistry routes, achieving precise control over the core–shell morphology. Electrochemical characterization through cyclic voltammetry demonstrated the intrinsic capability of rhodium to decompose H_2_O_2_ and generate oxygen. Beyond assessing the complete Au@Rh–ICG nanoplatform, we conducted independent cytotoxicity assays using pure gold nanoparticles, free ICG, and laser-activated ICG, which allowed us to isolate the contribution of each component and confirm the synergistic effects of the multifunctional system. The nanoplatform exhibited selective cytotoxicity, achieving 67% cell death in HOS osteosarcoma cells while affecting only 30% of healthy hFOB cells, thereby validating both its therapeutic potential and selective activity. Altogether, this study integrates materials science, electrochemistry, and cell biology approaches to present a promising theranostic system for osteosarcoma treatment.

## 2. Results

### 2.1. Optical Properties of Au@Rh Nanoparticles

**Figure 1:**
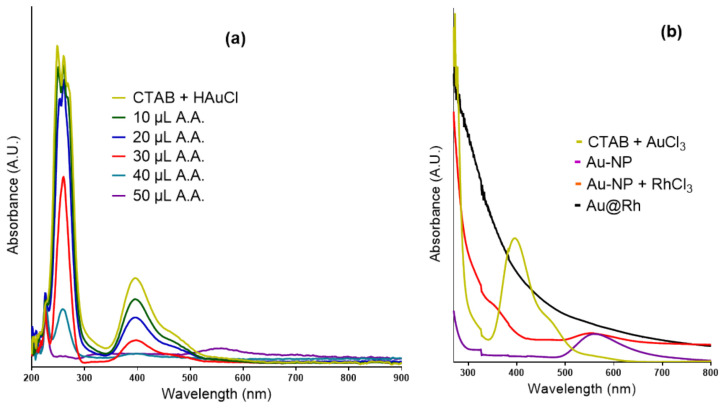
UV-Vis absorption profiles: (**a**) HAuCl_3_-CTAB solution during ascorbic acid (AA) titration, and (**b**) Rh shell growth stages on Au cores.

UV-Vis spectroscopy was employed to monitor nanoparticle growth due to its simplicity, reproducibility, and cost-effectiveness. The UV-Vis spectrum of the HAuCl_4_–CTAB solution displayed absorption maxima at 232 nm and 400 nm, corresponding to d–d electronic transitions of Au(III) ions. Upon gradual addition of ascorbic acid (AA), which reduced Au(III) ions, the precursor absorption bands diminished, and a surface plasmon resonance (SPR) peak emerged at 567 nm. This peak shifted to 541 nm upon complete reduction, indicating the formation of monodisperse, near-spherical Au cores, as evidenced by the stable SPR at 541 nm (Figure 1a).

Shell growth was monitored by tracking the deposition of a rhodium shell (Figure 1b). This was observed through the attenuation of the Au SPR signal (567 nm) following RhCl_3_ addition. The complete quenching of the Au SPR peak after NaBH_4_ reduction (black curve, Figure 1b) confirmed the full encapsulation of the Au cores by metallic Rh shells.

### 2.2. Structural Characterization of Au@Rh NPs

**Figure 2:**
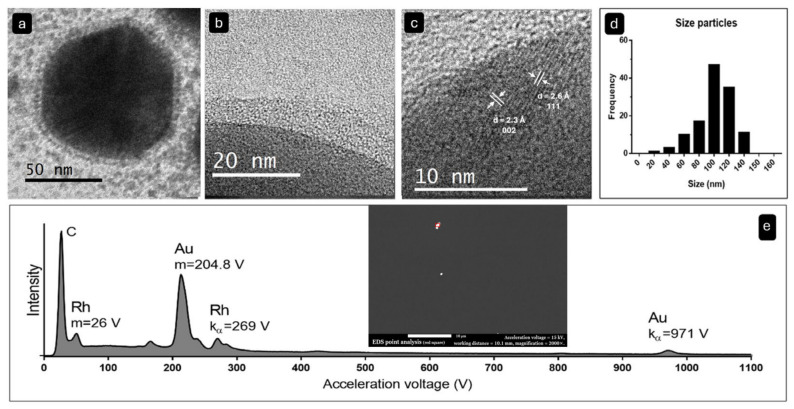
HRTEM/SEM-EDS of Au@Rh nanoparticles: (**a**) Core–shell architecture; (**b**) Core–shell interface; (**c**) Lattice spacing; (**d**) Size histogram; and (**e**) EDS spectrum.

The High-Resolution Transmission Electron Microscopy (HRTEM) (JEM-ARM200CF JEOL Ltd., Akishima, Tokyo, Japan) and Scanning Electron Microscopy–Energy-Dispersive X-ray Spectroscopy (SEM-EDS) (JSM-7800F JEOL Ltd., Akishima, Tokyo, Japan) analyses revealed spherical core–shell structures with an Au core radius of 90 ± 5 nm (higher density, Figure 2a,b), an Rh shell thickness of 15 ± 2 nm (lower density, Figure 2c), and an average particle diameter of ± 109 nm ± 7 nm (Gaussian distribution, Figure 2d). Crystallography: lattice fringes (Figure 2c) matched Au (111) (d = 2.35 Å) and (002) (d = 2.04 Å) planes (JCPDS 04-0784). Elemental Composition: EDS (15 kV) detected Au peaks at 2.048 eV (Mα) and 9.71 keV (Kα) and Rh peaks at 0.26 eV (Mα) and 2.69 keV (Kα). Carbon tape signals were excluded (Figure 2e).

### 2.3. Evaluation of Electrocatalytical Activity of AuRh NPs

**Figure 3:**
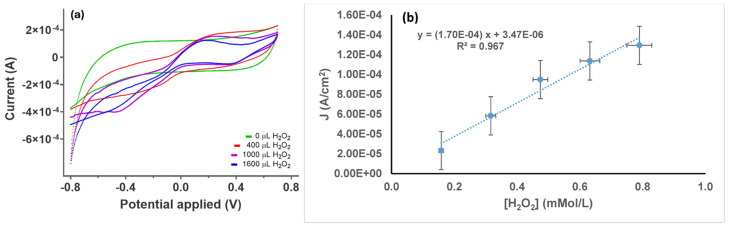
(**a**) Cyclic voltammetry of AuRh NPs with incremental H_2_O_2_; (**b**) Absolute calibration curve: Linear current density, J, vs. H_2_O_2_ standard concentration.

Cyclic voltammetry measurements performed in N_2_-saturated 0.1 M KOH revealed distinct electrochemical activity. An anodic peak at −0.2 V (vs. Ag/AgCl) was observed, corresponding to hydrogen peroxide (H_2_O_2_) oxidation, while a cathodic peak at −0.4 V indicated oxygen (O_2_) reduction (Figure 3a). The catalytic efficiency of the material was assessed by monitoring the anodic peak current density (I_p_), which increased linearly with H_2_O_2_ concentration, yielding a correlation coefficient of R^2^ > 0.98 (Figure 3b). This relationship followed the Randles–Ševčík equation [40]:

Ip=2.99×105·ne−·α12·A·C·D12·(v12)

where $n\left(e^{-}\right)$= electron transfer number, α = charge transfer coefficient (0.5), A = electroactive area, C = H_2_O_2_ concentration (mol/cm^3^), D = diffusion coefficient, and v = scan rate.

**Figure 4:**
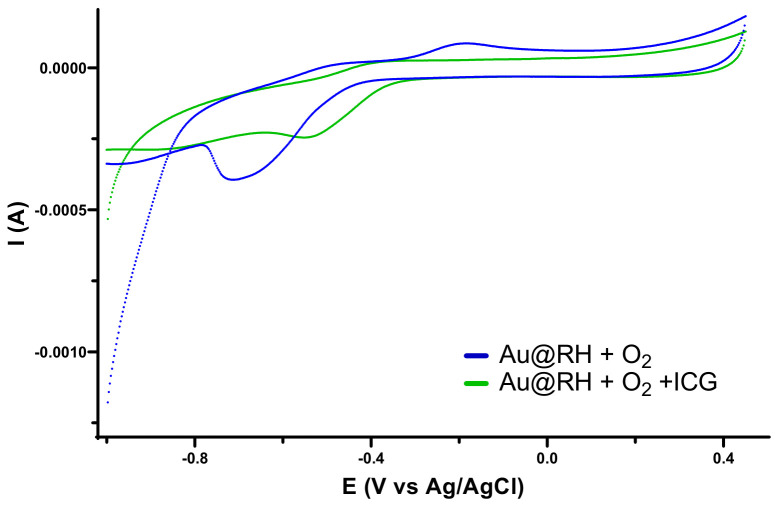
Cyclic voltammograms of AuRh NPs deposited on a vitreous carbon disk electrode in the absence and presence of indocyanine green (ICG), using 0.1 M phosphate buffer (pH 7.0) as the supporting electrolyte. The initial potential was −1.0 V vs. Ag/AgCl. The potential sweep rate was dE/dt = 10 mV/s.

Following the incorporation of indocyanine green (ICG), a transition from a quasi-reversible to an irreversible reaction was observed. This transition was indicated by the shift in the cathodic peak and the disappearance of the anodic peak once the applied potential reversed in the anodic direction (Figure 4). This demonstrates the binding of ICG to the Au@Rh nanoparticles comprising the working electrode and subsequent electron transfer, a critical phenomenon for ROS generation. Specifically, the oxygen reduction peak shifted to a less cathodic potential (E_p_ = −0.54 V), which is related to the formation of an Au@Rh-ICG-O_2_ complex that lowers the energy required for reduction. During the anodic sweep, the absence of the peroxide-to-oxygen oxidation peak suggests an irreversible EC (electrochemical–chemical) process, where an electroactive species (A) is reduced to an intermediate (B), which then undergoes a homogeneous chemical reaction to form an inactive product (C):

$$A+e^{-}⇌B$$

B⟶C

Such a case is defined as an EC reaction. It can thus be concluded that the presence of ICG within the electrolytic cell activates the Au@Rh nanoparticles, thereby promoting the complete oxidation of the hydrogen peroxide produced at the electrode–solution interface at a rate significantly higher than that dictated by the potential sweep conditions applied during the cyclic voltammetry tests shown in Figure 4.

### 2.4. Evaluation of Photothermal Conversion Efficiency

**Figure 5:**
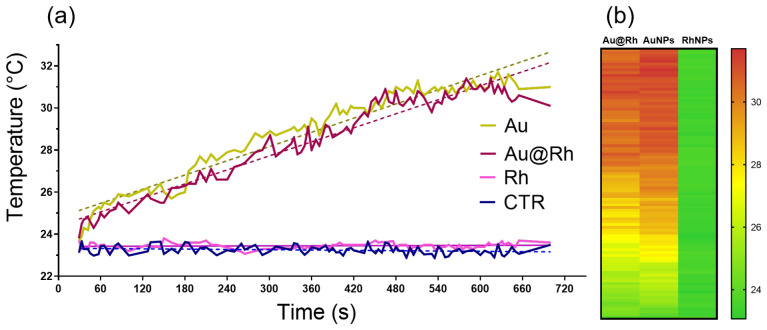
(**a**) Photothermal conversion of Au@Rh, AuNPs, and Rh nanoparticles under NIR irradiation (808 nm, 16.67 J/cm^2^). Temperature increase over time for Au@Rh, AuNPs, and Rh nanoparticles. The vehicle (absolute ethanol) was used as control (CTR). (**b**) Heat maps showing the temperature distribution after 10 min of irradiation for the indicated samples. CTR represents the control measurement obtained from the glass slide used as the sample support substrate.

The Au@Rh NPs exhibited a linear temperature increase upon NIR irradiation, comparable to the photothermal behavior observed in pure AuNPs, thereby confirming their photothermal conversion capability. Specifically, the temperature of Au@Rh NPs increased from 24 °C to 31 °C over 10 min, whereas pure AuNPs achieved the same temperature rise within 5 min using the vehicle (absolute ethanol) as a control. Pure Rh nanoparticles showed negligible photothermal conversion, confirming that the observed heating is primarily due to the gold core. Despite the difference in heating rates, both nanoparticle systems followed a similar linear heating trend, as illustrated in Figure 5a. Linear regression analysis yielded a correlation coefficient (R^2^ > 0.95), further validating the consistency and reliability of the photothermal response. The thermal distribution was visualized using a heat map (Figure 5b).

### 2.5. Cytotoxicity Assessment in Osteosarcoma (HOS) and Osteoblast (hFOB) Cells

**Figure 6:**
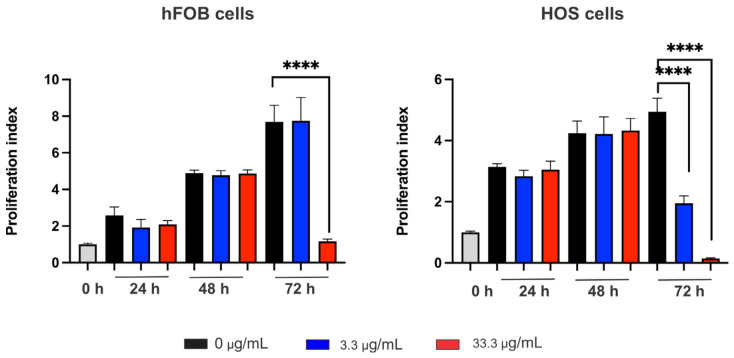
Relative proliferation index of hFOB and HOS cells exposed to AuNPs (3.3 and 33.33 μg/mL) for 24, 48 and 72 h, as determined by the MTT assay, Cells at the initial time point are indicated by the gray column. Data are presented as mean ± SEM (n = 3 independent experiments). Statistical analysis was performed using one-way ANOVA followed by Tukey’s multiple comparison test. **** *p* < 0.0001.

The effect of Au nanoparticles (AuNPs) on the proliferation of hFOB and HOS cells was evaluated at 0, 24, 48 and 72 h using concentrations of 3.3 and 33.3 μg/mL. As shown in Figure 6, AuNPs caused no major changes in hFOB or HOS cell proliferation at 24 and 48 h. However, after 72 h, 33.3 µg/mL significantly reduced cell viability, particularly in HOS cells, indicating a time- and concentration-dependent antiproliferative effect. In contrast, 3.3 µg/mL maintained relatively high proliferation levels.

**Figure 7:**
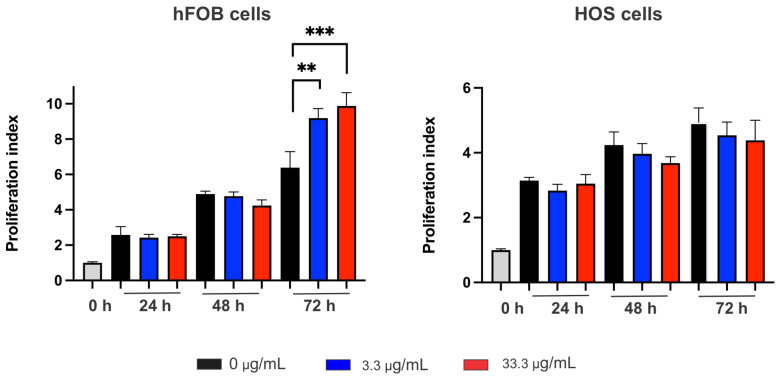
Relative proliferation index of hFOB and HOS cells exposed to Au@Rh core–shell nanoparticles (3.3 and 33.33 μg/mL) at 24, 48 and 72 h, as determined by the MTT assay, Cells at the initial time point are indicated by the gray column. Data are presented as mean ± SD (n = 3 independent experiments). Statistical analysis was performed using one-way ANOVA followed by Tukey’s multiple comparison test. ** *p* < 0.01, *** *p* < 0.001.

The effect of core–shell nanoparticles (Au@Rh NPs) on the proliferation of hFOB and HOS cells was evaluated at 0, 24, 48, and 72 h. Similarly to AuNPs, exposure to Au@Rh NPs at 24 and 48 h did not show significant changes. However, after 72 h of exposure, hFOB cells exhibited a slight increase in proliferation at 3.3 and 33.3 µg/mL, whereas HOS cell proliferation remained unchanged (Figure 7).

**Figure 8:**
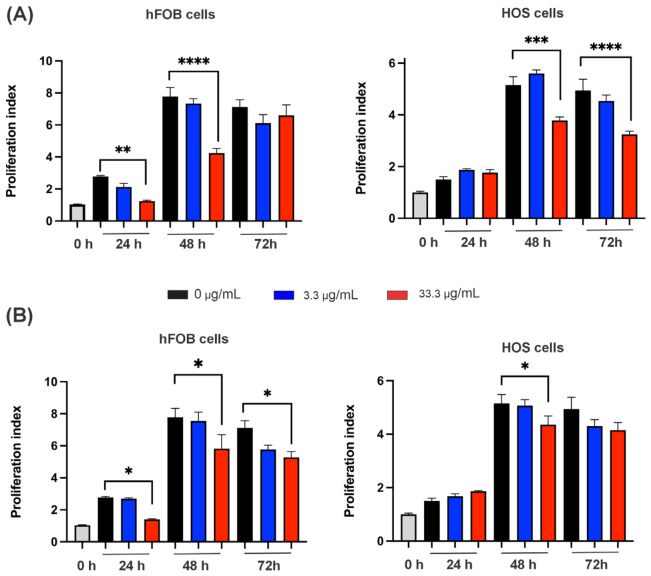
Relative proliferation index of hFOB and HOS cells exposed to (**A**) free ICG and (**B**) Au@Rh–ICG nanoparticles at concentrations of 3.3 and 33.33 μg/mL for 24, 48, and 72 h, as determined by the MTT assay, Cells at the initial time point are indicated by the gray column. Data are presented as mean ± SD (n = 3 independent experiments). Statistical analysis was performed using one-way ANOVA followed by Tukey’s multiple comparison test. Statistically significant differences compared to the control are indicated (* *p* < 0.05, ** *p* < 0.01, *** *p* < 0.001, **** *p* < 0.0001).

Independent cytotoxicity assays of the photosensitizer ICG were performed. As shown in Figure 8A, ICG alone induced only a slight reduction in the proliferation index of both hFOB and HOS cells, even after prolonged exposure times. In contrast, the Au@Rh–ICG nanoplatform (Figure 8B) exhibited a more pronounced time- and concentration-dependent antiproliferative effect, mainly observed at the highest concentration and more evident in hFOB cells during the early exposure periods. However, after 72 h of exposure, a marked decrease in proliferation was detected at 33.3 µg/mL, particularly in HOS osteosarcoma cells, whereas hFOB osteoblasts recovered their cell population. These findings suggest that coupling ICG to Au@Rh nanoparticles enhances the cytotoxic effect over time and promotes greater selectivity toward tumor cells.

**Figure 9:**
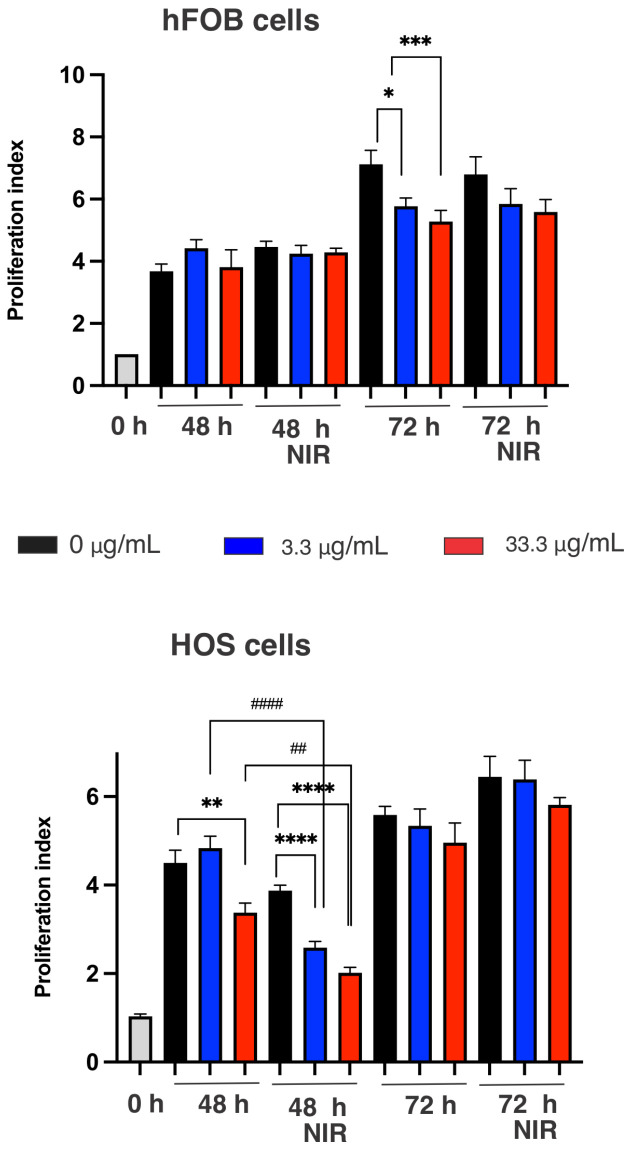
Relative proliferation index of hFOB and HOS cells treated with Au@Rh–ICG nanoparticles at concentrations of 3.3 and 33.33 μg/mL under dark conditions or upon near-infrared (808 nm) irradiation at 1 W for 10 min (20 J/cm^2^), evaluated at 24, 48, and 72 h post-treatment by the MTT assay, Cells at the initial time point are indicated by the gray column. Data are presented as mean ± SD (n = 3 independent experiments). Statistical analysis was performed using one-way ANOVA followed by Tukey’s multiple comparison test. Significant differences are indicated as follows: * *p* < 0.05, ** *p* < 0.01, *** *p* < 0.001, **** *p* < 0.0001 vs. control; ^##^ *p* < 0.01, ^####^ *p* < 0.0001 vs. non-irradiated cells.

Figure 9 illustrates the relative proliferation index of hFOB and HOS cells following treatment with Au@Rh–ICG nanoparticles (Au@Rh–ICG NPs) under dark conditions and after near-infrared (NIR) irradiation. Under dark conditions, the nanoparticles did not significantly affect the proliferation of either hFOB or HOS cells at any concentration or exposure time, confirming their cytocompatibility in the absence of external stimulation. In contrast, NIR irradiation (808 nm) induced a significant reduction in the proliferation index, particularly in HOS cells after 48 h of treatment, where the strongest antiproliferative effect was observed at 33.3 µg/mL. However, this effect was no longer evident at 72 h, suggesting partial recovery of cell proliferation over time. No significant differences were observed in irradiated hFOB osteoblasts, indicating a greater susceptibility of tumor cells to the photothermal treatment.

## 3. Discussion

The synthesis of gold nanoparticles via ascorbic acid (AA)-mediated reduction in AuCl_4_^−^ is well established, with AA facilitating electron transfer to reduce Au(III) to Au(0) through intermediate complexation [35]. In our system, CTAB plays a dual role: (1) as a surfactant stabilizing nascent Au nuclei via electrostatic interactions between CTA^+^ cations and AuCl_4_^−^ anions (forming [CTA]^+^-[AuCl_4_]^−^ complexes), and (2) as a structure-directing agent modulating nanoparticle growth kinetics through sub-/post-micellar effects [35]. UV-Vis spectroscopy confirmed the reduction process, marked by the disappearance of Au(III) d-orbital transitions (232 nm and 327 nm) and the emergence of a surface plasmon resonance (SPR) peak at 541 nm, characteristic of spherical Au cores (Figure 1a). The quenching of this SPR signal during Rh shell deposition (Figure 1b) aligns with prior reports of core–shell formation, where metallic shells attenuate plasmonic responses [32].

HRTEM analysis revealed polycrystalline Au cores with dominant (111) and (002) facets (JCPDS 04-0784), consistent with the fcc structure of gold (Figure 2c). The amorphous Rh shell (15 ± 2 nm thickness) lacked discernible lattice fringes, likely due to rapid NaBH_4_-driven reduction inhibiting atomic ordering, a phenomenon observed in other wet-chemical Rh nanostructure syntheses [34]. Spatially resolved EDS mapping confirmed the core–shell architecture, with distinct Au (Mα/Kα) and Rh (Mα/Kα) signals (Figure 2e).

### 3.1. Electrocatalytic Properties

The Rh shell exhibited pH-independent electrocatalytic activity for H_2_O_2_ decomposition, as demonstrated by cyclic voltammetry (CV) (Figure 3a and Figure 4). The linear relationship between anodic current density (I_p_) and H_2_O_2_ concentration (R^2^ > 0.98) follows the Randles–Sevcik equation, indicating diffusion-controlled kinetics [40]. The binding of ICG to Au@Rh nanoparticles on the working electrode is demonstrated by the rightward shift in the halfwave cathodic potential, which is associated with the reduction of O_2_ to H_2_O_2_. Moreover, the reversible anodic peak corresponding to the oxidation of H_2_O_2_ to O_2_ disappears completely. This behavior is characteristic of an irreversible EC (electrochemical–chemical) process, where the electroactive species (ICG) is chemically adsorbed onto the metal surface and subsequently undergoes a chemical reaction that prevents its reoxidation. The chemical adsorption is further evidenced by: (i) the absence of the oxygen reduction peak in the reverse sweep, and (ii) the dependence of the peak current on the scan rate, which is consistent with a surface-confined adsorbed species. Consequently, ICG is not encapsulated within a carrier but rather firmly attached to the external surface of the Au@Rh nanoparticles. Conventional encapsulation efficiency and release kinetics measurements (e.g., dialysis methods) are therefore not applicable. Under physiological conditions (pH = 7.4, 37 °C), no significant ICG desorption is expected during the 24 h cell exposure period, as the chemical bond requires extreme conditions (highly acidic pH or elevated potentials) to be broken. This strong and stable adsorption ensures that the photosensitizer remains associated with the nanoparticles throughout the cytotoxicity experiments.

This demonstrates that spacer molecules or biconjugates anchored to the surface of the metal nanoparticles are not required. Additionally, the integration of ICG within the nanoparticle structure serves to initiate the decomposition of hydrogen peroxide produced at the electrode–solution interface.

### 3.2. Cytocompatibility and Therapeutic Implications

The physicochemical properties of gold nanoparticles (AuNPs), particularly their size and shape, play a critical role in determining their cytotoxicity. Previous studies have shown that spherical AuNPs within the 6–22 nm range do not compromise the viability of hFOB or MG-63 cells [41]. Woźniak et al. systematically compared five different shapes and sizes of AuNPs, demonstrating that small spherical particles (~10 nm) are the most cytotoxic, while larger particles (160–370 nm) exhibit significantly lower toxicity [42]. Our spherical Au@Rh nanoparticles (~109 nm) fall within this low-toxicity range, and the concentrations used (up to 33.33 µg/mL, ~0.17 µM) are far below the toxic thresholds reported for large particles (300 µM). Thus, the absence of basal cytotoxicity in our system is consistent with the literature and attributable to both large size and spherical morphology.

Nevertheless, after 72 h, the highest concentration tested induced a reduction in cell proliferation in both cell lines, suggesting a time- and concentration-dependent effect. This is an important finding, since there are very limited studies evaluating the long-term cytotoxicity of AuNPs. These results suggest that spherical AuNPs can retain their biocompatibility even at larger sizes, although mainly during short-term exposure.

Furthermore, Au@Rh nanostructures exhibited a favorable cytocompatibility profile at short exposure times, with no significant reduction in cell viability at the tested concentrations after 24 and 48 h. This behavior may be attributed to the formation of a RhOx surface layer under physiological conditions, which could enhance biocompatibility while the metallic Rh core preserves its catalytic properties. Interestingly, at 72 h, an increase in the proliferation index was observed in hFOB cells. However, this result should be interpreted with caution, since the MTT assay reflects metabolic activity rather than a direct increase in cell number.

To further enhance functionality, Au@Rh nanoparticles were integrated with indocyanine green (ICG), a compound originally developed as a dye and later approved by the FDA as a contrast agent for microsurgical procedures and as a photosensitizer, thereby generating a multifunctional platform. In our study, ICG alone did not induce cytotoxic effects in HOS or hFOB cells after 24–48 h at a concentration of 3.3 µg/mL. However, higher concentrations and prolonged exposure (72 h) resulted in decreased cell viability. These findings are consistent with previous reports indicating that ICG concentrations below 15 µg/mL are generally well tolerated by osteoblasts [43] and MG-63 osteosarcoma cells [44].

Importantly, the slight cytotoxic effects observed under dark conditions, together with the significant reduction in cell proliferation upon NIR irradiation, mainly in HOS cells, highlight the controlled and stimulus-responsive behavior of the Au@Rh–ICG system during short-term exposure (48 h). The enhanced sensitivity of HOS osteosarcoma cells compared to non-tumoral hFOB cells suggests a degree of selectivity that is highly desirable for therapeutic applications. However, these findings were observed only at short exposure times, and further long-term studies are required to evaluate the safety of the system. This selectivity may be associated with the reduced capacity of cancer cells to manage oxidative stress and hyperthermia-induced damage, as well as their altered redox homeostasis. Although similar phototherapeutic effects have been reported in other cancer models, such as breast cancer [45,46], it is important to consider the intrinsic biological differences among tumor types. Osteosarcoma arises from mesenchymal tissue, whereas breast cancer is of epithelial origin, involving distinct signaling pathways and tumor microenvironment characteristics. In this context, the differential response observed here may be further influenced by the unique metabolic features of osteosarcoma cells, including their redox imbalance and susceptibility to oxidative stress, which can potentiate both photothermal and photodynamic effects.

Overall, these findings support the potential of the Au@Rh–ICG nanoplatform as a biocompatible and effective system for NIR-triggered phototherapy, particularly in the context of osteosarcoma treatment. In addition, the results highlight the promise of rhodium-based nanomaterials in catalytic nanomedicine applications [34], where their intrinsic catalytic activity may further enhance therapeutic outcomes.

Nevertheless, despite these encouraging results, additional studies are required to better understand nanoparticle penetration, distribution, and retention within the dense extracellular matrix characteristic of osteosarcoma tumors. These aspects are critical for optimizing therapeutic efficacy in vivo.

Taken together, the Au@Rh–ICG nanosystem represents a significant step forward in the development of multifunctional nanoplatforms for the treatment of hypoxic and metabolically complex tumors, providing a promising strategy for improving the effectiveness of phototherapy in osteosarcoma, enabling controlled drug release, and improving photoacoustic imaging performance [32].

## 4. Materials and Methods

### 4.1. Synthesis of Au@Rh Core–Shell Nanoparticles

Metal nanoparticle synthesis was performed using hydrogen tetrachloroaurate(III) hydrate (HAuCl_4_·xH_2_O, Sigma-Aldrich-520918, Saint Louis, MO, USA) and rhodium(III) chloride hydrate (RhCl_3_·xH_2_O, Sigma-Aldrich-520772) as ionic precursors, with ascorbic acid (Merck, Darmstadt, Germany) and sodium borohydride (NaBH_4_, Fluka-71321, Seelze, Germany) as reducing agents. Cetyltrimethylammonium bromide (CTAB, Sigma-Aldrich-H5882) served as the surfactant. All reagents were used as received without further purification. Seed Formation: 8 mL of 100 mM CTAB solution was heated to 60 °C in a water bath. Under vigorous magnetic stirring (500 rpm), 250 µL of 10 mM HAuCl_4_ was added, followed by 200 µL of 80 mM ascorbic acid, inducing a pink coloration (Au core formation). Shell Deposition: 850 µL of 10 mM RhCl_3_ was introduced, with continuous stirring for 5 min. Then, 400 µL of 100 mM NaBH_4_ was added to initiate Rh shell growth, evidenced by a color shift to dark gray. Purification: Particles were washed with ethanol (1:1 *v*/*v*), centrifuged (15,000× *g*, 10 min), and redispersed via ultrasonication (30 s, 40 kHz). This wash cycle was repeated twice, and the final product was suspended in 1 mL of absolute ethanol and stored at 4 °C. A schematic representation of the two-step synthesis is shown in Figure S2.

### 4.2. Spectroscopic Characterization of Au@Rh Core–Shell Nanoparticles

UV-Vis spectra (400–800 nm) were acquired using a PerkinElmer XLS spectrophotometer (PerkinElmer, Shelton, CT, USA) to monitor plasmon resonance shifts during Au core growth. Morphological analysis was conducted on a JEOL JSM 7800F microscope (JEOL, Tokyo, Japan, 15 kV, secondary/backscattered electron detection). Atomic-resolution TEM was performed on a JEOL JEM-ARM 200CF (20 kV) with EDS for elemental mapping. The average core radius, shell thickness, and overall particle diameter were determined by measuring 50 nanoparticles from three independent syntheses using HRTEM images. Size distribution analysis was performed with ImageJ2 software v2.x.x. The dilution used was 1:50,000 in absolute ethanol with prior sonication (40 kHz, 30 s) to ensure adequate dispersion and avoid aggregates. The size histogram (Figure 2d) was constructed from these values, yielding a mean diameter of 109 ± 7 nm.

### 4.3. Photothermal Conversion: Experimental Setup

Photothermal measurements were performed using a portable infrared (IR) camera (Model HTI-19, HT Instruments, Guangzhou, China) with the following specifications: Spectral range: 8–14 μm (far-infrared band). Thermal resolution: ≤0.1 °C (at 30 °C). Accuracy: ±2 °C or ±2% of reading (whichever is greater). Field of view (FOV): 25° × 19° with a distance-to-spot ratio (D:S) of 10:1. Temperature range: −20 °C to +400 °C (extendable to +1000 °C with an optional filter). Sensitivity (NETD): <50 mK. Refresh rate: 9 Hz. Adjustable emissivity: 0.1–1.0 (pre-calibrated for metallic surfaces and aqueous media).

Sample preparation: An ethanol suspension of nanoparticles was deposited on a glass slide to achieve a uniform distribution of 100 µg Au@Rh over an area of 0.196 cm^2^ (equivalent to a 0.5 cm diameter spot). The samples were dried in a vacuum oven at 60 °C for 60 min. An identical protocol was applied to pure gold nanoparticles (AuNPs), synthesized under the same conditions but without the rhodium shell.

Irradiation: Samples were exposed to an 808 nm near-infrared (NIR) laser at 1 W power, delivering a fluence of 16.67 J/cm^2^ over a 10 min period. Temperature changes were recorded with a temporal resolution of 3 s intervals.

Environmental control: All experiments were conducted at 23 °C and 50% relative humidity inside a laminar flow chamber to minimize thermal drift and ensure consistent experimental conditions. Absolute ethanol was used as a control in both cases.

### 4.4. Electrochemical Characterization

Electrocatalytic activity was evaluated using cyclic voltammetry (CV) on a Metrohm Autolab 302N potentiostat (Metrohm, Herisau, Switzerland). Working electrode: Glassy carbon modified with Au@Rh ink prepared by mixing 10 µL of nanoparticle suspension, 250 µL isopropanol, and 10 µL of Nafion, followed by sonication for 3 min. Reference electrode: Ag/AgCl (3 M KCl). Counter electrode: Pt wire. Parameters: Scan range: +0.8 to −0.7 V vs. Ag/AgCl; electrolyte: pH 7.4 phosphate buffer.

For FRA impedance analysis at OCP, a DRP-C110 screen-printed carbon electrode was used (working and counter electrodes: carbon; reference electrode: silver). The electrolyte was phosphate-buffered saline, pH 7.4.

### 4.5. Formation of the Nanostructured System Au@Rh-ICG

Indocyanine green (ICG) was used as the photosensitizing agent. Prior to use, the vial was subjected to a drying process at 50 °C for 60 min. Subsequently, 1 mg of the dried compound was transferred into a microtube and resuspended in 1 mL of a dimethyl sulfoxide/deionized water mixture (1:4). The ICG solution was prepared immediately before use, as storage in solution is not feasible due to dimer formation, precipitation, and alterations in its optical properties. To facilitate integration with the metallic nanostructures, 5 mg of Au@Rh core–shell nanoparticles were added to the ICG solution. The mixture was stirred at room temperature for 60 min and then centrifuged at 13,000× *g* for 5 min. The supernatant was discarded, and 0.5 mL of absolute ethanol was added to the pellet, followed by 5 min of sonication. This washing step was repeated until no residual ICG remained. The resulting nanocomposite, suspended in saline solution (0.9% NaCl, PISA Laboratory, Guadalajara, Jalisco, Mexico), was dispensed into pre-weighed microtubes in 50 µL aliquots. The suspension mass was recorded, after which the material was dried at 50 °C for 3 h under vacuum. The final weight of each microtube containing the dried material was used to determine the mass corresponding to 50 µL of suspension, enabling calculation of the concentration of the Au@Rh–ICG nanocomposite.

### 4.6. Cytotoxicity Assessment in hFOB and HOS Cells

The human osteoblast cell line hFOB1.19 (CRL-3602) and human osteosarcoma cell line HOS (CRL-1543) were acquired from the American Type Culture Collection (ATCC, Manassas, VA, USA) and cultured in Eagle Minimum Essential Medium (12800-058 GIBCO, Auckland, New Zealand) supplemented with 10% fetal bovine serum (FBS) (S1560-500 Biowest, Riverside, MO, USA) and 1% penicillin–streptomycin (15240-062 GIBCO, Grand Island, NY, USA) in a humidified incubator at 37 °C and 5% CO_2_. Once the cells reached a confluency of about 80%, they were trypsinized and seeded in 96-well plates at a density of 5 × 10^3^ cells/well and incubated for 24 h. Cells were treated with AuNPs, Au@Rh and Au@Rh-ICG nanoparticles at concentrations of 3.33 or 33.33 µg/mL in fresh medium and incubated for 0, 24, or 48 h. After incubation, the medium was replaced with 200 µL of MTT solution (5 mg/mL in culture medium). Following a 3 h incubation period, the resulting formazan crystals were dissolved by adding 200 µL of DMSO per well, with gentle agitation for 5 min. Absorbance was measured at 570 nm (signal) and 660 nm (reference) using a Biotek Epoch microplate reader. Untreated cells served as positive controls, and blank wells were used for background correction. All experiments were conducted in triplicate. Colloidal stability of Au@Rh nanoparticles in PBS and absolute ethanol was assessed by UV-Vis spectroscopy; detailed data are presented in Supplementary Document S3 (Figure S3).

### 4.7. Photosensitiser Activation

Two 96-well plates were prepared under the conditions described, divided into three groups (0, 3.3, and 33.33 µg/mL of Au@Rh-ICG). A subgroup of each concentration was irradiated with an 808 nm NIR laser (1 W, fluence 16.67 J/cm^2^) for a period of 10 min. The selection of these irradiation parameters was based on photothermotoxicity assays using hFOB cell cultures, in which potential changes were evaluated at 5, 10, and 15 min under laser irradiation conditions with a wavelength of 808 nm, a power of 1 W, and positioned over an area of 0.196 cm^2^ from the samples (see Figure S1 in the Supplementary Information section). No statistically significant differences in cell viability were observed between 10 and 15 min; therefore, 10 min was chosen to standardize the protocol and minimize handling time outside the incubator. After this, the MTT assay was conducted after 24 h and 48 h for Au@Rh-ICG and pure ICG.

## 5. Conclusions

A multifunctional Au@Rh core–shell nanoplatform functionalized with indocyanine green (ICG) was successfully developed and evaluated as a theranostic strategy for osteosarcoma. The system combines the photothermal properties of gold with the electrocatalytic activity of rhodium, enabling hydrogen peroxide decomposition and local oxygen generation to mitigate tumor hypoxia.

Electrochemical characterization confirmed the catalytic role of the rhodium shell, while photothermal assays demonstrated efficient near-infrared light-to-heat conversion. Cytotoxicity studies showed that AuNPs, Au@Rh nanoparticles, and free ICG were biocompatible in both HOS osteosarcoma and hFOB osteoblast cells. Importantly, upon NIR irradiation, the complete Au@Rh–ICG system induced a selective photodynamic effect, achieving up to 67% cytotoxicity in HOS cells while preserving higher viability in normal osteoblasts.

Overall, these results highlight the potential of Au@Rh–ICG nanoparticles as a multifunctional platform that integrates catalysis, photothermal conversion, and photodynamic activity, offering a promising approach to overcome hypoxia-related limitations in osteosarcoma therapy. In terms of future work, we plan to conduct further experiments to elucidate the mechanisms underlying the selective cytotoxicity and oxygen-generating capacity of our Au@Rh ICG nanoplatform. Specifically, we will quantify intracellular reactive oxygen species (ROS) levels using fluorescent probes, and evaluate the expression of heat shock proteins (HSP70 and HSP90) and antioxidant enzymes (catalase and superoxide dismutase) using Western blotting and quantitative PCR (qPCR). These studies will help clarify whether the reduced capacity for managing oxidative stress in osteosarcoma cells contributes to the observed selectivity.

## Data Availability

The original contributions presented in this study are included in the article/Supplementary Materials. Further inquiries can be directed at the corresponding author.

## Supplementary Materials

The following supporting information can be downloaded at: https://www.mdpi.com/article/10.3390/ijms27146253/s1.

## Abbreviations

The following abbreviations are used in this manuscript: OS: Osteosarcoma; PDT: Photodynamic Therapy; PS: Photosensitizer; ROS: Reactive Oxygen Species; NIR: Near-Infrared; NPs: Nanoparticles; AuNPs: Gold Nanoparticles; Au@Rh: Gold–Rhodium Core–Shell Nanoparticles; ICG: Indocyanine Green; Au@Rh-ICG: Gold–Rhodium Core–Shell Nanoparticles functionalized with Indocyanine Green; HOS: Human Osteosarcoma Cell Line (ATCC^®^ CRL-1543™); hFOB: Human Fetal Osteoblast Cell Line (ATCC^®^ CRL-11372™); EPR: Enhanced Permeability and Retention Effect; CV: Cyclic Voltammetry; SPR: Surface Plasmon Resonance; HRTEM: High-Resolution Transmission Electron Microscopy; SEM-EDS: Scanning Electron Microscopy with Energy-Dispersive X-ray Spectroscopy; MTT: 3-(4,5-Dimethylthiazol-2-yl)-2,5-diphenyltetrazolium bromide assay.
