# Supplementary material for: Study of the Cytotoxic Effects of Au@Rh Core–Shell Metal Particles on the Osteosarcoma Cell Line HOS and the hFOB Osteoblast Cell Line
Source: Int J Mol Sci. 2026 Jul 14;27(14):6253. doi: 10.3390/ijms27146253 (PMC13409995; doi:10.3390/ijms27146253)
Supplement: Supplementary file 1 [file ijms-27-06253-s001.zip › ijms-4291997-supplementary.pdf]

International Journal of *Molecular Sciences*

MDPI

Supporting Information

Study of the Cytotoxic Effects of Au@Rh Core–Shell Metal Particles on the Osteosarcoma Cell Line HOS and the hFOB Osteoblast Cell Line

Sergio Zamudio-Lucero [1,2,3], Martín Trejo-Valdez [2,*], Nury Pérez-Hernández [3], Ángel Bañuelos-Hernández [3] and María Elena Manríquez-Ramírez [2]

[1] Unidad Profesional Interdisciplinaria de Biotecnología (UPIBI), Instituto Politécnico Nacional (IPN), Av. Acueducto, La Laguna Ticomán, Gustavo A. Madero, Ciudad de México 07340, Mexico; szamudiol@ipn.mx

[2] Escuela Superior de Ingeniería Química e Industrias Extractivas (ESIQIE), Instituto Politécnico Nacional (IPN), Zacatenco, Edificio 8 1er. Piso, Ciudad de México 07300, Mexico; mmanriquez@ipn.mx

[3] Escuela Nacional de Medicina y Homeopatía (ENMH), Instituto Politécnico Nacional (IPN), Av. Guillermo Massieu Helguera 239, La Escalera, Gustavo A. Madero, Ciudad de México 07320, Mexico; nperezh@ipn.mx (N.P.-H.); abanuelosh@ipn.mx (A.B.-H.)

* Correspondence: mtrejov@ipn.mx

S1. Cytotoxicity Assessment Conditioons using hFOBCells

Preliminary photothermocytotoxicity assays were performed using hFOB cell cultures, and potential changes were evaluated at 5, 10, and 15 minutes of irradiation (808 nm, 1 W, 0.196 cm^2). The laser fluence (LF) of irradiated samples was calculated by applying the following formula:

$$LF = \frac{\text{Power (W)} \times \text{time (s)}}{\text{Area (cm}^2\text{)}} = \frac{1\text{ W} \times 600\text{ s}}{0.196\text{ cm}^2} \approx 16.6\text{ J/cm}^2$$

No statistically significant differences in cell viability were observed between 10 and 15 minutes; therefore, 10 minutes was selected to standardize the protocol and minimize handling time outside the incubator (see Figure S1).

Infrared dosimetry	
**Time (s)**	**Energy (J/cm^2)**
**0**	0
**300**	8.33
**600**	16.67
**900**	25.0

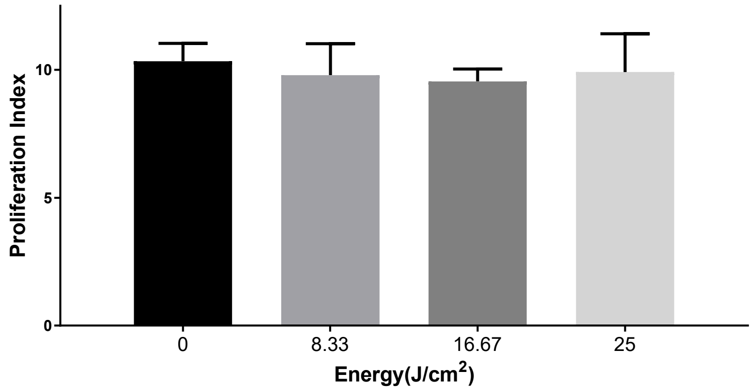

Figure S1. Infrared dosimeter assays performed on hFOB cells by using an 808 nm infrared laser (808 nm, 1 W, 0.196 cm^2).

S2. The schematic representation of the methodology for synthesising core-shell nanoparticles.

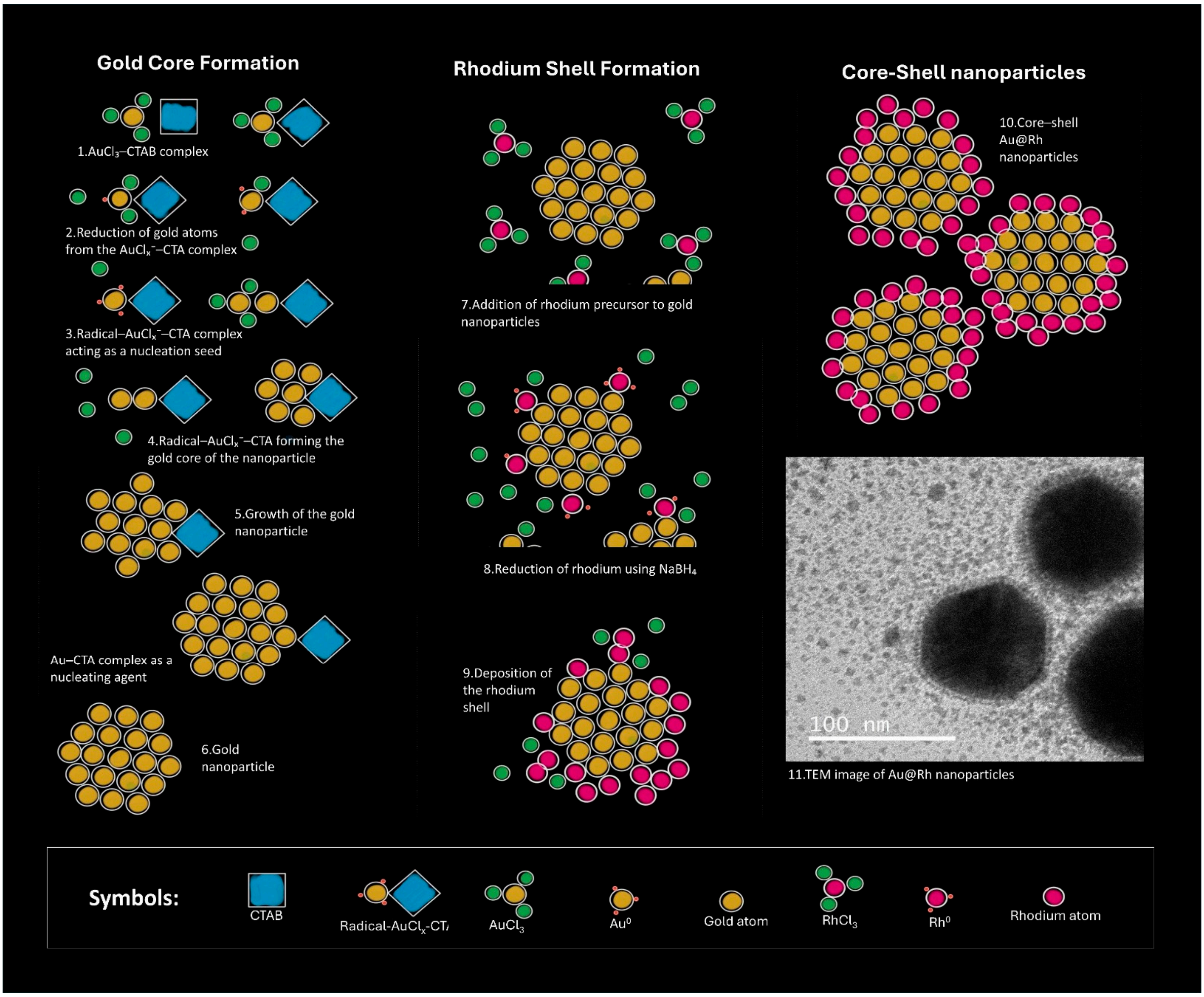

Figure S2. A Schematic representation of the synthesis of Au@Rh core-shell nanoparticles. A TEM image of the final nanoparticle is shown in the inset. Adapted from the mechanism proposed by Khan et al. [1].

As illustrated in Figure S2, the process of gold core formation of nanoparticles is delineated in steps 1–6:

1. Dissolution of CTAB in water.
2. Addition of $HAuCl_4$ to form the $AuCl_3$–CTAB complex.
3. Introduction of ascorbic acid (AA) as reducing agent.
4. Reduction of Au(III) to Au(0) via a radical intermediate, forming nucleation seeds.
5. Growth of the seeds into larger gold nanoparticles.
6. Final spherical Au core (approximately 90 nm diameter).

The process of Rhodium shell formation is delineated in steps 7–11::

7. Addition of $RhCl_3$ to the Au core suspension.

8. Complexation of Rh(III) ions on the gold surface.

9. Reduction with $NaBH_4$.

10. Deposition of metallic rhodium as a shell around the gold core.

11. Final Au@Rh core-shell nanoparticle (total diameter ~109 nm; shell thickness ~15 nm).

S3. Colloidal stability study of Au@Rh nanoparticles

A **logarithmic concentration range** was used to cover the concentrations employed in the main paper, including a higher concentration of 100 μg/mL. At 4 h, the SPR peak was centered at 545 nm in both media, with no detectable shift, indicating good monodispersion. After 24 h, the SPR peak shifted to 552 nm in ethanol and to 554 nm in PBS, accompanied by moderate broadening, indicating partial nanoparticle aggregation. The greater shift in PBS is likely due to charge screening.

All cell experiments were initiated within 4 h of nanoparticle preparation. Therefore, during the critical initial exposure period (first 4 h), the nanoparticles remained well dispersed. The partial aggregation observed after 24 h did not affect cell viability, as confirmed by the cytotoxicity assays (Figures 6–8 of the main manuscript). For future in vivo studies, biofunctionalization (e.g., cell-membrane coating) will be implemented to improve long-term colloidal stability, as previously reported [2].

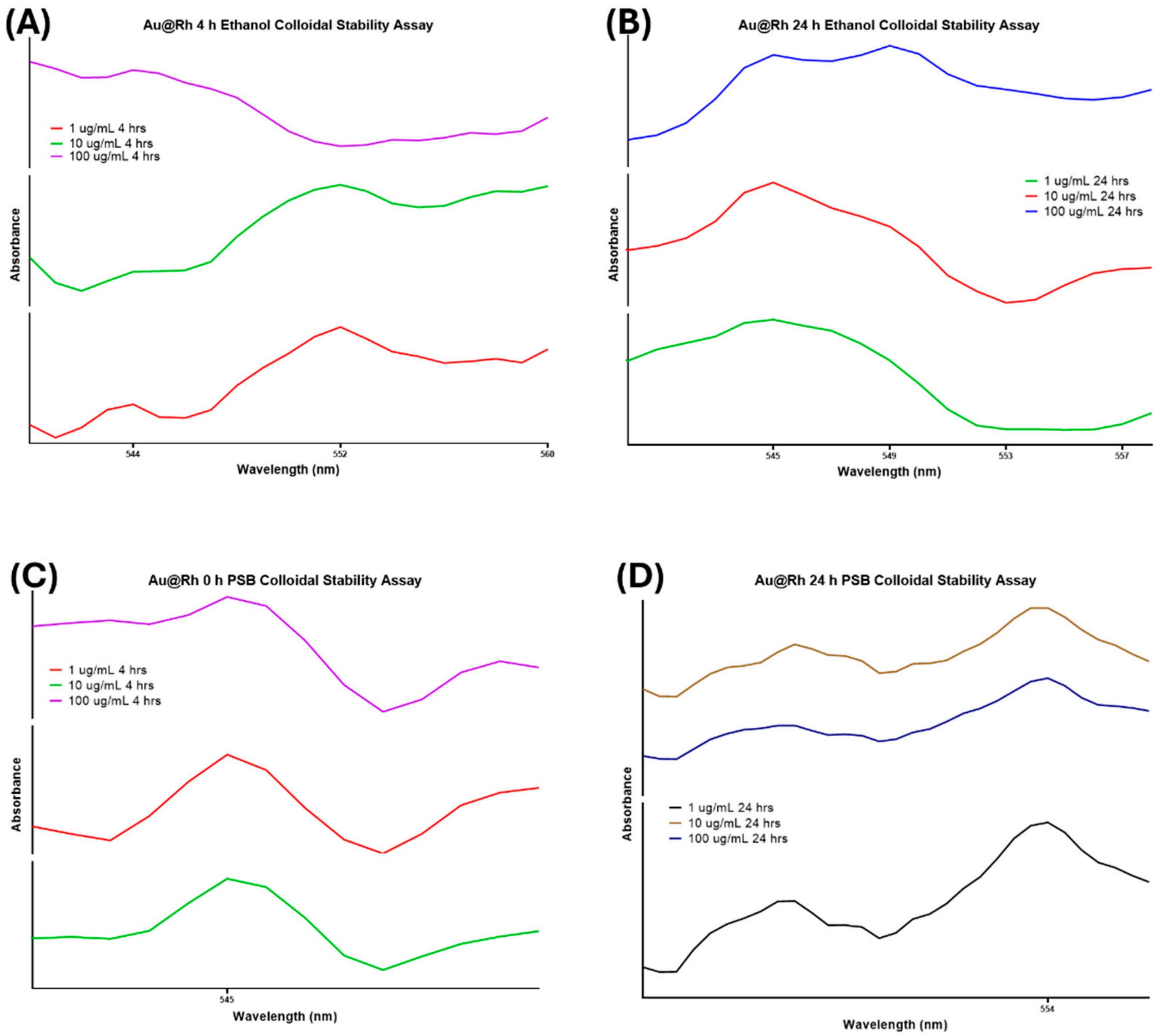

Figure S3. UV-Vis absorption spectra of Au@Rh nanoparticles at 1, 10, and 100 μg/mL (the highest concentration used in cell experiments was 33.3 μg/mL). Spectra were recorded in **(A)** absolute ethanol after 4 h, **(B)** absolute ethanol after 24 h, **(C)** PBS (pH 7.4) after 4 h, and **(D)** PBS after 24 h. The spectra at 0 h were identical to those at 4 h and are not shown. The surface plasmon resonance (SPR) peak position and width were monitored to assess nanoparticle aggregation.

References

1. Khan, Z., Singh, T., Hussain, J. I., & Hashmi, A. A. Au(III)–CTAB reduction by ascorbic acid: Preparation and characterization of gold nanoparticles. Colloids Surf B Biointerfaces 2012, *104*, 11-17. https://doi.org/10.1016/j.colsurfb.2012.11.017
2. Wang J, et al. A Porous Au@Rh Bimetallic Core-Shell Nanostructure as an H_2O_2-Driven Oxygenerator to Alleviate Tumor Hypoxia for Simultaneous Bimodal Imaging and Enhanced Photodynamic Therapy. Adv Mater 2020, *32*(22), e2001862. doi: 10.1002/adma.202001862.
